# Diagnostic Accuracy of a Telemedicine-Based Shoulder Examination Compared With MRI in Rotator Cuff Syndrome: A Cross-Sectional Study

**DOI:** 10.7759/cureus.99527

**Published:** 2025-12-18

**Authors:** Rodrigo A Beraldo, Leonardo Zanesco, Pedro Henrique S da Cruz, Mauro E Gracitelli, Jorge Assunção, Caio Checchia, Fernando B Andrade-Silva, Eduardo A Malavolta

**Affiliations:** 1 Orthopaedics and Traumatology, Instituto Jundiaiense de Ortopedia e Traumatologia, Jundiai, BRA; 2 Orthopaedics and Traumatology, Hospital das Clínicas da Faculdade de Medicina da Universidade de São Paulo, São Paulo, BRA; 3 Orthopaedics and Traumatology, Diagnóstico das Américas S.A. Hospital 9 de Julho, São Paulo, BRA; 4 Shoulder and Elbow Unit, Hospital Sírio-Libanês, São Paulo, BRA; 5 Orthopaedics and Traumatology, Hospital do Coração (HCor), São Paulo, BRA

**Keywords:** diagnostic test accuracy, mri images, rotator cuff, shoulder examination, telemedicine

## Abstract

Background

Telemedicine is increasingly used in musculoskeletal care, but the accuracy of remote physical examination for rotator cuff syndrome, compared with magnetic resonance imaging (MRI), remains unclear. This study evaluated the diagnostic performance of telemedicine-based shoulder maneuvers against MRI, with secondary analyses including in-person examination.

Methods

A cross-sectional diagnostic accuracy study was conducted. Patients underwent standardized teleconsultations with Jobe, infraspinatus, and Bear Hug tests, each analyzed for pain and weakness. All patients also underwent the same in-person maneuvers. MRI of the affected shoulder, reviewed by blinded musculoskeletal radiologists, served as the reference standard. Sensitivity, specificity, positive predictive value (PPV), negative predictive value (NPV), and balanced accuracy were calculated. Secondary analyses included comparisons between telemedicine and in-person performance, and agreement between modalities.

Results

Thirty-two patients (mean age, 59.6 years) were included. Against MRI, telemedicine tests were highly sensitive: Jobe pain sensitivity was 96.2%, infraspinatus weakness sensitivity was 76.9%, and Bear Hug weakness sensitivity was 57.1%. In-person tests showed similar sensitivity for most maneuvers, with a tendency to classify fewer tendons as weak in MRI-negative shoulders. Paired analyses demonstrated minor differences for pain outcomes, but significant differences for weakness, with telemedicine being more sensitive and in-person examinations being more conservative in classifying weakness. Agreement between modalities was excellent for pain (κ up to 1.00) and moderate for weakness (κ, 0.11-0.47).

Conclusion

Telemedicine-based shoulder examination demonstrated high sensitivity compared with MRI, similar to in-person examination. Because all patients had MRI-confirmed pathology, and few true negative tendons were available, the ability of either modality to rule out rotator cuff tears based solely on clinical testing was limited. These findings support the use of telemedicine as a sensitive triage tool, while MRI remains necessary for definitive diagnosis and treatment planning.

## Introduction

Rotator cuff disorders are among the most frequent shoulder pathologies and represent a significant cause of musculoskeletal disability [1]. Population-based studies have shown that rotator cuff tears affect approximately 20%-22% of adults, with prevalence increasing markedly with age [2,3]. Symptomatic tears are common in individuals over 60 years, while asymptomatic tears are often detected in imaging studies of the general population [4]. Recent reviews confirm that rotator cuff disease affects 6.8%-22.4% of adults older than 40 [5]. Given this high prevalence and clinical impact, improving diagnosis and management strategies is critical. Concurrently, telemedicine has rapidly expanded, offering new possibilities for musculoskeletal assessment and potentially improving access to specialized care [6].

Physical examination remains the cornerstone of rotator cuff assessment in traditional outpatient settings, although its diagnostic accuracy varies across maneuvers. Recent efforts have evaluated whether these examinations can be reliably reproduced remotely. Telemedicine consultations may achieve diagnostic accuracy and reproducibility comparable to in-person assessments, with high patient satisfaction [7,8]. Tele-rehabilitation programs have also demonstrated improved function and pain in rotator cuff patients [9]. Newer approaches, such as the Bari Shoulder Telemedicine Examination Protocol (B-STEP) standardized protocol for telemedicine examination, and studies validating remote measurement of strength and range of motion, support the feasibility of structured remote shoulder assessment [10,11].

Despite these advances, in-person and remote examinations remain limited by poor specificity compared to magnetic resonance imaging (MRI). Physical tests often yield false positives, since structural abnormalities are commonly observed in asymptomatic individuals, leading to discrepancies between clinical and imaging findings [12,13]. Moreover, remote evaluation adds further challenges, such as variability in positioning and the absence of palpation. These limitations underscore the need to determine how well telemedicine-based examinations correspond with MRI findings.

The primary objective of this study was to evaluate the diagnostic performance of telemedicine-based shoulder examination maneuvers against MRI findings in patients with rotator cuff syndrome, with sensitivity as the main diagnostic accuracy parameter. As a secondary objective, we compared telemedicine and in-person examinations performed in the same patients, focusing on agreement between modalities.

We hypothesized that telemedicine-based examination would demonstrate high sensitivity for MRI-confirmed tendon tears and substantial agreement with in-person examination.

## Materials and methods

Study design

This prospective cross-sectional diagnostic accuracy study aimed to assess the performance of standardized telemedicine-based physical examination maneuvers in patients with rotator cuff syndrome, using MRI as the reference standard. The protocol was approved by the Institutional Ethics Committee of Hospital das Clínicas da Faculdade de Medicina da Universidade de São Paulo, São Paulo, Brazil (51184821.4.0000.0068) and conducted in accordance with the principles of the Declaration of Helsinki. The investigation was reported following the Standards for Reporting Diagnostic Accuracy Studies (STARD) guidelines.

Study population and eligibility criteria

Patients were recruited consecutively from a specialized shoulder center. Eligible participants were adults older than 30 years with at least two months of shoulder pain and MRI evidence of rotator cuff pathology (tendinopathy or partial/full-thickness tears). Exclusion criteria included prior surgery on the affected shoulder, other ipsilateral upper-limb disorders that could interfere with evaluation (such as osteoarthritis, neurologic conditions, infection, or post-traumatic sequelae), and inability to perform the standardized maneuvers due to cognitive or sensory impairment. The study population represents the telemedicine arm of a previously conducted diagnostic accuracy study by our group, which evaluated the reliability and reproducibility of teleconsultation for rotator cuff syndrome [8].

Clinical examination protocols

All patients underwent a telemedicine-based and an in-person physical examination on the same day. The teleconsultations were conducted by the principal investigator, who did not participate in any of the in-person evaluations. In-person examinations were performed by shoulder surgeons at our institution, each with more than 10 years of clinical experience. Thus, no patient was evaluated by the same examiner in both modalities. Both telemedicine and in-person examiners were blinded to MRI findings at the time of the assessments.

For the telemedicine examination, patients received an instructional video before the consultation, explaining positioning, camera setup, and execution of the required maneuvers. During the teleconsultation, the examiner supervised the process in real time and provided corrective feedback to ensure adequate execution. Resistance was applied using the contralateral limb.

For the in-person examination, the same maneuvers were performed with direct examiner-applied resistance and standardized definitions of positivity.

The three maneuvers assessed in both modalities were Jobe’s test (supraspinatus) [14], the infraspinatus external-rotation resistance test [15], and the Bear Hug test (subscapularis) [16]. Each maneuver was analyzed separately for pain and for weakness. Pain positivity was defined as patient-reported discomfort during the maneuver. Weakness was defined as the inability to maintain the tested position against resistance (contralateral arm for telemedicine, examiner-applied for in-person).

The order of examinations was randomized: half of the patients underwent in-person evaluation first, followed by telemedicine, while the other half underwent telemedicine first, followed by in-person examination. Both assessments were performed on the same day. Randomization was performed consecutively according to patient arrival order, alternating between starting with telemedicine or in-person examination.

MRI assessment

All patients underwent shoulder MRI using a 1.5-Tesla scanner with T1- and T2-weighted sequences in coronal oblique, sagittal oblique, and axial planes. Images were interpreted by experienced musculoskeletal radiologists blinded to telemedicine and in-person findings. Each tendon (supraspinatus, infraspinatus, subscapularis) was categorized as normal, tendinopathy, or tear (partial or full thickness). For diagnostic accuracy analyses, pain outcomes considered any MRI-detected pathology (tendinopathy or full-thickness tear) as a positive reference standard. For weakness outcomes, only full-thickness tears were considered MRI-positive. 

Variables and outcomes

The primary outcome was the diagnostic accuracy of telemedicine-based tests compared with MRI. Secondary outcomes included the diagnostic accuracy of in-person tests compared with MRI, paired differences in sensitivity between telemedicine and in-person examinations, and the agreement between modalities, regardless of MRI findings.

Sample size justification

This was an exploratory diagnostic accuracy study, and no formal a priori sample size calculation was performed. The final sample (n = 32) included all consecutive eligible patients recruited during the study period at a single specialized shoulder center.

To justify the adequacy of this number, Buderer’s formula [17] for diagnostic test studies was applied, assuming an expected sensitivity (S) of 0.90, disease prevalence (P) of 0.80, a 95% confidence level (Z = 1.96), and an allowable error (d) of 0.13. Although this allowable error is slightly higher than in larger confirmatory studies, it is acceptable for exploratory or pilot diagnostic accuracy investigations conducted within real-world recruitment constraints. Under these assumptions, approximately 26 patients would be required to estimate sensitivity with acceptable precision, indicating that our final sample of 32 patients exceeded the minimum requirement.

We acknowledge that other studies evaluating similar outcomes have included larger samples, often twice as large. However, these studies were designed for different purposes - typically to test non-inferiority or to assess multiple diagnostic endpoints - whereas the present study aimed primarily to explore the feasibility and sensitivity of telemedicine-based maneuvers. The consecutive sampling strategy, limited recruitment window, and pragmatic nature of telemedicine implementation also contributed to the smaller, but adequate, sample size.

Statistical analysis

Sensitivity was the primary diagnostic accuracy parameter and was calculated with 95% confidence intervals using the Wilson method for each tendon-test pairing. Positive predictive value (PPV) and negative predictive value (NPV) were also calculated descriptively. Accuracy was defined as the proportion of correctly classified observations, calculated as $\frac{TP + TN}{TP + FP + TN + FN}$. Given the very small number of true negative tendons - particularly for pain-based outcomes - specificity and related measures were not emphasized and were not used as primary outcomes. For paired comparisons of sensitivity between modalities, McNemar’s test was applied, and the corresponding χ² statistics and p-values were reported. Agreement between telemedicine and in-person findings was summarized using percentage agreement and Cohen’s kappa [18], each with 95% confidence intervals. All analyses were descriptive and were not powered for formal non-inferiority testing.

## Results

Baseline characteristics

Thirty-two patients were included, with a mean age of 59.6 years (SD 6.7); 71.8% were female, and 90.6% had right-hand dominance. The right shoulder was affected in 78.1% of cases, and the mean duration of symptoms was 46.9 months (SD 34.1). Pain at rest was reported by 71.9% of patients, while 96.9% complained of night pain. The prevalence of comorbidities was 28.1% for diabetes, 62.5% for hypertension, 34.4% for hypercholesterolemia, and 18.8% for thyroid disease, while 9.4% were active smokers (Table 1).

**Table 1:** Baseline demographic and clinical characteristics of patients Continuous variables are presented as mean (standard deviation), and categorical variables as absolute frequencies and percentages.

Characteristic	Total (n = 32)
Age, mean (SD), years	59.6 (6.7)
Female, n (%)	23 (71.8%)
Dominant side affected, n (%)	23 (71.8%)
Duration of pain, mean (SD), months	46.9 (34.1)
Pain at rest, n (%)	23 (71.9%)
Night pain, n (%)	31 (96.9%)
Smoker, n (%)	3 (9.4%)
Diabetes, n (%)	9 (28.1%)
Thyroid disease, n (%)	6 (18.8%)
Hypertension, n (%)	20 (62.5%)
Hypercholesterolemia, n (%)	11 (34.4%)

MRI findings

MRI demonstrated that all patients had supraspinatus pathology (100%), consisting of supraspinatus tendinopathy in 18.8% and full-thickness tears in 81.2%. Infraspinatus pathology was present in 93.8% of patients, including tendinopathy in 53.1% and full-thickness tears in 40.6%. Subscapularis pathology was identified in 90.6% of patients, with tendinopathy in 68.7% and full-thickness tears in 21.9%. No partial-thickness tears were observed in this sample (Table 2).

**Table 2:** MRI characteristics of rotator cuff lesions “Pathology” includes tendinopathy or any type of tear. No partial-thickness tears were identified in this cohort; the category is included for methodological completeness. MRI: magnetic resonance imaging

Tendon (n = 32 shoulders)	Pathology n (%)	Tendinopathy n (%)	Partial-thickness n (%)	Full-thickness n (%)
Supraspinatus	32 (100%)	6 (18.8%)	0 (0%)	26 (81.2%)
Infraspinatus	30 (93.8%)	17 (53.1%)	0 (0%)	13 (40.6%)
Subscapularis	29 (90.6%)	22 (68.7%)	0 (0%)	7 (21.9%)

Telemedicine and in-person physical examination

In teleconsultations, the Jobe, infraspinatus, and Bear Hug tests were positive for pain in 96.9%, 90.6%, and 75.0% of patients, respectively, and for weakness in 90.6%, 56.2%, and 37.5%, respectively (Table 3).

**Table 3:** Telemedicine and in-person physical examination findings Values represent the number and percentage of patients with positive results for each maneuver, analyzed separately for pain and weakness.

Test	Telemed (n = 32)	In-person (n = 32)
Jobe pain, n (%)	31 (96.9%)	31 (96.9%)
Jobe weakness, n (%)	29 (90.6%)	26 (81.2%)
Infraspinatus pain, n (%)	29 (90.6%)	27 (84.3%)
Infraspinatus weakness, n (%)	18 (56.2%)	9 (28.1%)
Bear Hug pain, n (%)	24 (75.0%)	20 (62.5%)
Bear Hug weakness, n (%)	12 (37.5%)	5 (15.7%)

Because weakness outcomes were analyzed only in tendons with MRI-confirmed full-thickness tears, the denominators for weakness differ from those for pain outcomes. These subgroup sizes are explicitly reported in the MRI findings section.

The comparison of telemedicine-based examination against MRI demonstrated consistently high sensitivity across maneuvers. For example, Jobe's pain showed 96.2% sensitivity, and infraspinatus weakness achieved 76.9% sensitivity. Detailed diagnostic accuracy results for both telemedicine and in-person examinations are presented in Table 4. Because our sample included only patients with MRI-confirmed rotator cuff pathology at the shoulder level and very few true negative tendons, specificity estimates for pain-based tests were not emphasized.

**Table 4:** Diagnostic accuracy of telemedicine and in-person physical examination against MRI Sensitivity, PPV, NPV, and accuracy were calculated for each tendon-test pairing using MRI as the reference standard. Specificity values are reported descriptively but not emphasized due to the small number of true negative tendons. MRI, magnetic resonance imaging; PPV, positive predictive value; NPV, negative predictive value; CI, confidence interval

Test vs MRI	Sensitivity (%)		Specificity (%)		PPV (%)		NPV (%)		Accuracy (%)	
	Telemed	In-person	Telemed	In-person	Telemed	In-person	Telemed	In-person	Telemed	In-person
Jobe pain	96.2	96.2	0.0	0.0	80.6	80.6	0.0	0.0	48.1	48.1
Jobe weakness	88.5	88.5	0.0	66.7	79.3	88.5	0.0	66.7	44.2	77.6
Infraspinatus pain	100.0	92.3	15.8	26.3	44.8	48.0	100.0	83.3	57.9	59.3
Infraspinatus weakness	76.9	46.2	57.9	84.2	55.6	66.7	78.6	70.6	67.4	65.2
Bear Hug pain	85.7	57.1	28.0	36.0	25.0	26.7	87.5	66.7	56.9	46.6
Bear Hug weakness	57.1	57.1	68.0	96.0	33.3	80.0	85.0	87.5	62.6	76.6

In-person testing generally yielded similar sensitivity to telemedicine for most maneuvers, but with fewer MRI-negative tendons being classified as weak, particularly in Bear Hug weakness assessments.

Paired comparisons revealed similar sensitivity for Jobe tests and higher sensitivity for telemedicine in infraspinatus weakness and Bear Hug pain. For weakness maneuvers, in-person examinations more frequently classify tendons as strong, despite MRI-confirmed pathology. Significant paired differences were found for infraspinatus weakness (χ² = 5.78, p = 0.016) and Bear Hug weakness (χ² = 5.18, p = 0.023), as summarized in Table 5.

**Table 5:** Paired differences between telemedicine and in-person examination (telemed - in-person) McNemar’s test was used for paired comparisons, and the corresponding chi-square (χ²) values and p-values are presented.

Test	Sens in-person	Sens telemed	ΔSens	χ²	p
Jobe pain	96.2%	96.2%	0.0%	-	NA
Jobe weakness	88.5%	88.5%	0.0%	-	NA
Infraspinatus pain	92.3%	100%	+7.7%	-	NA
Infraspinatus weakness	46.2%	76.9%	+30.8%	5.78	0.016
Bear Hug pain	57.1%	85.7%	+28.6%	-	NA
Bear Hug weakness	57.1%	57.1%	0.0%	5.18	0.023

Agreement between modalities was excellent for pain (κ up to 1.00) and moderate for weakness (κ = 0.11-0.47), as shown in Table 6.

**Table 6:** Agreement between telemedicine and in-person examinations Agreement between telemedicine-based and in-person examination results for each shoulder maneuver, expressed as percentage agreement and Cohen’s kappa coefficients with 95% confidence intervals. Interpretation of kappa values: <0.00 poor; 0.00-0.20 slight; 0.21-0.40 fair; 0.41-0.60 moderate; 0.61-0.80 substantial; 0.81-1.00 almost perfect agreement.

Test	% Agreement	Kappa
Jobe pain	100%	1.00
Jobe weakness	78.1%	0.11
Infraspinatus pain	84.4%	0.37
Infraspinatus weakness	65.6%	0.35
Bear Hug pain	87.5%	0.71
Bear Hug weakness	78.1%	0.47

## Discussion

This study demonstrated that telemedicine-based physical examination for rotator cuff syndrome provides high sensitivity compared to MRI. Jobe and infraspinatus pain tests reached sensitivities above 90%, and infraspinatus weakness also showed high sensitivity. However, it is essential to note that clinical-radiological correlation is not perfect, even for in-person examinations. Previous studies have shown that shoulder physical tests, compared with MRI or arthroscopy, present variable sensitivity and are frequently positive even when structural lesions are absent or clinically irrelevant. Thus, the diagnostic performance of telemedicine should be interpreted in light of the inherent limitations of clinical shoulder testing in general [12,13].

Our results corroborate and extend previous evidence. In our prior study, telemedicine shoulder examinations for rotator cuff syndrome were reliable and reproducible, with high patient satisfaction and substantial agreement with in-person assessments [8]. However, when MRI is used as the reference, the present study and previous reports indicate that the accuracy of individual maneuvers is limited. Bradley et al. [7] found that while some telehealth shoulder tests achieved moderate agreement with MRI, certain maneuvers still showed limitations in diagnostic performance when compared with imaging.

Other studies have reinforced this discrepancy. Wang et al. [19] reported that while the range of motion can be consistently evaluated remotely, maneuvers assessing strength or instability are less reliable in telemedicine settings. Similarly, Lamplot et al. [20] emphasized that although most physical tests can be adapted to virtual care, the lack of examiner-applied resistance impairs the validity of strength-based maneuvers. In contrast, Rabin et al. [21] observed higher agreement between telemedicine and in-person assessments for shoulder disorders. However, their study relied on clinical impression rather than MRI confirmation, which may explain the more favorable results.

When in-person examinations were compared against MRI, the overall pattern remained similar in terms of sensitivity. For weakness outcomes, in-person testing tended to identify fewer shoulders as weak, which is expected when examiner-applied resistance is used. These findings corroborate the literature on physical shoulder testing, which describes improved discrimination between symptomatic and asymptomatic shoulders when maneuvers are performed with direct examiner-applied resistance [22]. Nonetheless, even in-person examinations are affected by the high prevalence of imaging abnormalities in asymptomatic individuals, and clinical tests frequently yield positive findings in shoulders with structural changes that may not be clinically relevant [12,13,23].

Direct paired comparisons between modalities provided further insights. Telemedicine showed higher sensitivity, particularly for infraspinatus weakness. Statistically significant differences were observed for infraspinatus and Bear Hug weakness. Agreement between modalities was excellent for pain, with kappa values indicating almost perfect concordance, but only moderate for weakness outcomes. Similar observations have been reported in the telemedicine literature: pain-related maneuvers are generally reproducible in remote assessments, whereas strength- and instability-related tests remain less consistent due to the absence of examiner-applied resistance [24,25].

This study has several limitations. The most important limitation is that all included patients had MRI evidence of rotator cuff pathology at the shoulder level, with no control group of individuals without disease. This spectrum effect results in very few true negative tendons, especially for supraspinatus tests, and makes specificity and NPV difficult to interpret. For this reason, we chose not to emphasize specificity in our analyses, particularly for pain-based outcomes, and focused instead on sensitivity and agreement between modalities.

Second, it was a single-center analysis with a modest sample size, which resulted in wide confidence intervals for some estimates - particularly specificity - and no formal power to test non-inferiority.

Third, our definition of a positive reference standard differed by outcome (tendinopathy or partial tears were considered positive for pain outcomes, while only full-thickness tears were considered positive for weakness outcomes). This approach reflects clinical reasoning but may have inflated sensitivity for pain, while reducing comparability across maneuvers. Additionally, the assumption that all patients with full-thickness tears necessarily present weakness is debatable, as clinical experience shows that some individuals may retain preserved strength despite structural discontinuity. This discordance between anatomy and function could have further influenced the accuracy estimates for weakness outcomes.

Fourth, MRI was used as the reference standard rather than arthroscopy. Although examinations followed a standardized 1.5-T protocol and were interpreted by experienced musculoskeletal radiologists blinded to clinical data, inter-reader reliability was not assessed, and misclassification of tendon status cannot be excluded. Moreover, previous studies have demonstrated that arthroscopy provides superior accuracy compared with MRI in evaluating rotator cuff tears [26,27], supporting the view that MRI may underestimate tendon lesions.

Fifth, the conduct of the index tests differed inherently between modalities: telemedicine relied on patient-applied resistance and camera positioning, whereas in-person testing used examiner-applied resistance. Moreover, teleconsultations were all performed by the principal investigator, while other experienced shoulder surgeons conducted in-person examinations. Although this design minimized intra-examiner bias, it introduced potential inter-examiner variability. Nevertheless, in our previous study [8], a subgroup analysis in which two in-person examinations were performed independently on the same patients by the principal investigator and other surgeons demonstrated very high concordance, mitigating this concern. It should also be acknowledged that different telemedicine models exist, and a trained assistant or healthcare professional beside the patient could improve test validity, although at the cost of reducing the practicality and scalability of remote consultations.

Finally, although concerns about sequence or fatigue effects could arise, our protocol included randomization of the order of examinations: half of the patients underwent in-person assessment first, followed by telemedicine, while the other half underwent the reverse order. This design helped to mitigate order-related bias. Other limitations include the restricted set of maneuvers evaluated, the absence of objective quantification of strength (e.g., dynamometry), and the fact that the sample was drawn from a specialized shoulder center, where patients had chronic symptoms and an MRI was required. Therefore, external validity to other contexts - such as primary care, acute presentations, or less standardized teleconsultations - may be limited.

Despite these limitations, the study has important strengths. All patients underwent both examination modalities, allowing for paired analyses rarely reported in the literature. The protocol was standardized, MRI interpretation was blinded, and separate examiners performed the two types of assessment, minimizing bias. Few studies have evaluated paired examinations in the same patients, which provides a unique within-subject comparison and strengthens the validity of our findings.

Future research should build on these results by incorporating objective tools to enhance remote strength assessment, such as handheld dynamometers, wearable sensors, or artificial intelligence-based motion analysis. Larger, multicenter studies could validate these findings across different clinical settings, while independent assessments by multiple examiners would improve reproducibility. Such approaches may help telemedicine evolve from a sensitive triage tool into a comprehensive diagnostic strategy for rotator cuff disorders, potentially expanding access to specialized musculoskeletal care.

## Conclusions

Telemedicine-based physical examination for rotator cuff syndrome demonstrated consistently high sensitivity when compared with MRI. Because true-negative tendons were uncommon in this cohort, specificity estimates were not reliable and should not be interpreted as primary diagnostic outcomes. Telemedicine, therefore, represents a feasible, highly sensitive triage tool for initial assessment of rotator cuff disorders, while MRI remains essential for definitive characterization and treatment planning.
